# Editorial: Improving the quality of outcome measurement for adults with disabilities receiving community-based services

**DOI:** 10.3389/fresc.2023.1163522

**Published:** 2023-03-31

**Authors:** Renáta Tichá, Brian Abery, Jan Šiška

**Affiliations:** ^1^ Institute on Community Integration, University of Minnesota Twin Cities, St. Paul, MN, United States; ^2^ Faculty of Education, Charles University, Prague, Czechia

**Keywords:** outcome measurement, service quality, quality of life, disability, community-based

**Editorial on the Research Topic** Improving the quality of outcome measurement for adults with disabilities receiving community-based services

Most of us, regardless of whether we have a disability, desire to live lives that are characterized as being of high quality. The extent to which people with disabilities are able to live the types of lives they desire is often far more dependent on the availability and effectiveness of the paid and unpaid support they receive from others than for the general population. The capacity to monitor the extent to which the quality of life of people with disabilities reflects their personal goals and dreams and is comparable to that of individuals without disabilities is critical if we are to understand the extent to which community-based services are doing what they are intended to do. Outcome measures are needed that are person-centered and longitudinal to assess various aspects of life as well as the quality of support service recipients receive. These measures need to be sufficiently sensitive to change that the impact of policy, funding, and programmatic changes on the outcomes people experience can be determined over time. They would also preferably have the capacity to be used with different disability populations who receive community support, including people with intellectual and developmental disabilities (IDD), physical disabilities, mental health challenges, traumatic brain injury (TBI)/acquired brain injury (ABI), and age-related conditions.

To be confident that outcome measures associated with community-based services can adequately assess both quality of services and the outcomes people with disabilities experience, data are needed with respect to their reliability, validity, and sensitivity to change. Indicators of quality and unmet support needs as directly perceived by service recipients must be considered paramount when developing, administering, and interpreting results based on these measures. Attempts to formulate frameworks to guide measure development and measure evaluation have not been restricted to the United States and have been underway in many countries for some time now. However, there has been limited collaboration between measure developers internationally.

This special issue of **Frontiers in Rehabilitation Sciences: Disability, Rehabilitation, and Inclusion** is designed to fill this gap in understanding the current landscape of measurement approaches used to assess the quality of services and life outcomes of adults with disabilities in the context of community-based services and support. In this special issue on *Improving the Quality of Outcome Measurement for Adults with Disabilities Receiving Community-Based Services*, we focus on different components and approaches to outcome measurement in 12 original articles from the United States and worldwide.

Utilizing a global perspective, Swenson provided a historical and philosophical context for outcome measurement targeting people with disabilities. She reminded us of the importance of measuring the outcomes from a human rights perspective, understanding that outcome measurement is inherently holoscopic or carefully focused on a certain aspect of the person's functioning or support and therefore in many ways biased.

Several articles highlighted the importance of measuring the quality of community-based services using specific outcome measures. Using data from the US-developed Personal Outcome Measures and Basic Assurances, Friedman investigated how the quality of service provision at different levels (individual, organizational, and environmental) contributes to personal outcomes people with IDD. Bradley and Hiersteiner provided a historical overview of the US-based National Core Indicators-IDD In Person Survey, a tool that most US states use to measure service quality and point out the need for periodic evaluation of such measures to determine their continued utility and validity.

In Ireland, Burke et al. reported on using the Personal Outcome Scale with people with intellectual disabilities (IDs) receiving services in community-based settings to examine the psychometric properties of the measures and the quality of life outcomes experienced by this population.

Articles from Norway, Germany, and the United States point to the shortcomings of the current outcome measurement approaches to service quality. Tøssebro et al. reported the results of their study in Norway on the motivations for outcome measurement, highlighting administrative needs and demands and the ambiguous impact of such measurement on service quality. Rohrmann and Schaedler discussed outcome measurement for people with disabilities in the context of Germany's rather rigid system of services and propose to conceptualize quality assessment as “local quality dialogues for collective learning.” Riesen et al. pointed out the inadequacies of traditional outcome measures when assessing employment outcomes for people with the most significant disabilities.

Several articles provide concrete suggestions for modernizing and improving outcome measurement for people with disabilities. In the United States, Bogenschutz et al. reflected on their Virginia Costs and Outcomes Project to point out the importance of utilizing existing linked large datasets, using advanced data analytic techniques, and including the voices of people with disabilities themselves for a comprehensive measurement approach. Caldwell and Machledt made policy-guided recommendations on improving outcome measurement in the context of Home and Community Based Services (HCBS), including establishing a regular stakeholder input mechanism, improving the approach to data collection, and requiring transparent public reporting. Roberts and Abery discussed the historical absence of person-centered approaches to measuring the outcomes of people with disabilities, largely due to the application of the medical model to this population. They described the importance of and ways in which measures can and have recently been designed to reflect the person's experiences with and perspectives on their services and life outcomes.

Two articles take on specific topics within the theme of outcome measurement. Beadle-Brown et al. reported on the results of their mapping of outcome measures of service quality onto transition domains for youth with disabilities. Houseworth et al. discussed the role of risk adjustment in HCBS outcome measurement, identified commonly used risk adjustors, and proposed risk adjustors for consideration when measuring the outcomes of people with disabilities to increase measurement precision.

The topics of this special issue were authored by professionals with extensive experience in policy and practice across different service systems and contributed to the field of outcome measurement in several ways. Some articles focused on defining service quality and the life outcomes people with disabilities experience as part of a broader community. Other articles provided a historical, geographic, and/or policy context for outcome measurement and pointed to existing issues and areas for needed improvement. These include the need for person-centered measures , are capable of being used longitudinally and have adequate sensitivity to change, can be used with multiple disability populations, and possess sufficient psychometric precision (i.e., reliability and validity) to be used in the context of their intended decision-making contexts, and minimize bias.

